# Supplementary material for: Development of a high dimensional imaging mass cytometry panel to investigate spatial organization of tissue microenvironment in formalin-fixed archival clinical tissues
Source: Heliyon. 2024 May 14;10(10):e31191. doi: 10.1016/j.heliyon.2024.e31191 (PMC11128903; doi:10.1016/j.heliyon.2024.e31191)

All supplementary information

**Supplementary Table 1.** Overview of tissue types (control tissue micro array) used in cTMA construction

| Normal liver | Normal heart | Normal spleen | Normal kidney | Normal breast | Placenta |  |
| --- | --- | --- | --- | --- | --- | --- |
| Lung adenocarcinoma, normal side | Lung adenocarcinoma, tumor side | Oral squamous cell carcinoma, high a11 expression 1 | Oral squamous cell carcinoma, high a11 expression 2 | Mammary carcinoma 1 | Mammary carcinoma 2 |  |
| Mammary carcinoma 3 | Oral squamous cell carcinoma HPV positive | Pancreas carcinoma, tumor center | Pancreas carcinoma, margin towards colon | Rectum carcinoma, margin fat and nerves | Rectum carcinoma, tumor side |  |
| Osteosarcoma 1 | Osteosarcoma 2 | Melanoma, margin, skin | Melanoma, tumor side | Normal pancreas | Normal brain |  |
|  |  |  |  |  |  |  |
| Tonsil 1 | Tonsil 2 | Inflamed appendix | Liver carcinoma, tumor center | Liver carcinoma, tumor front | Normal liver |  |
|  |  |  |  |  |  |  |
| Tongue squamous cell carcinoma 1 | Tongue squamous cell carcinoma 2 | Tongue squamous cell carcinoma 3 | Tongue squamous cell carcinoma 4 | Glioma 1 | Glioma 2 |  |
|  |  |  |  |  |  |  |

Table S2 Clinical parameters of patients in tTMA

| Patient ID | Age | Gender | Treatment | TNM | Alcohol | Smoking | 5-year survival* | Recurrence |
| --- | --- | --- | --- | --- | --- | --- | --- | --- |
| Patient 1 | 68 | F | Surgery + Radiotherapy | T2N2M0 | No | No | Yes | No |
| Patient 2 | 57 | M | Surgery + Radiotherapy | T4N2M0 | No | No | Yes | No |
| Patient 3 | 61 | M | Surgery + Radiotherapy | T2N1M0 | No | No | Yes | No |
| Patient 4 | 56 | M | Surgery + Radiotherapy | T2N2M0 | No | Yes | Yes | No |
| Patient 5 | 53 | M | Surgery + Radiotherapy | T1N2M0 | No | Yes | Yes | Yes |

*All patients were alive 5 years after primary treatment.

Supplementary figure 1


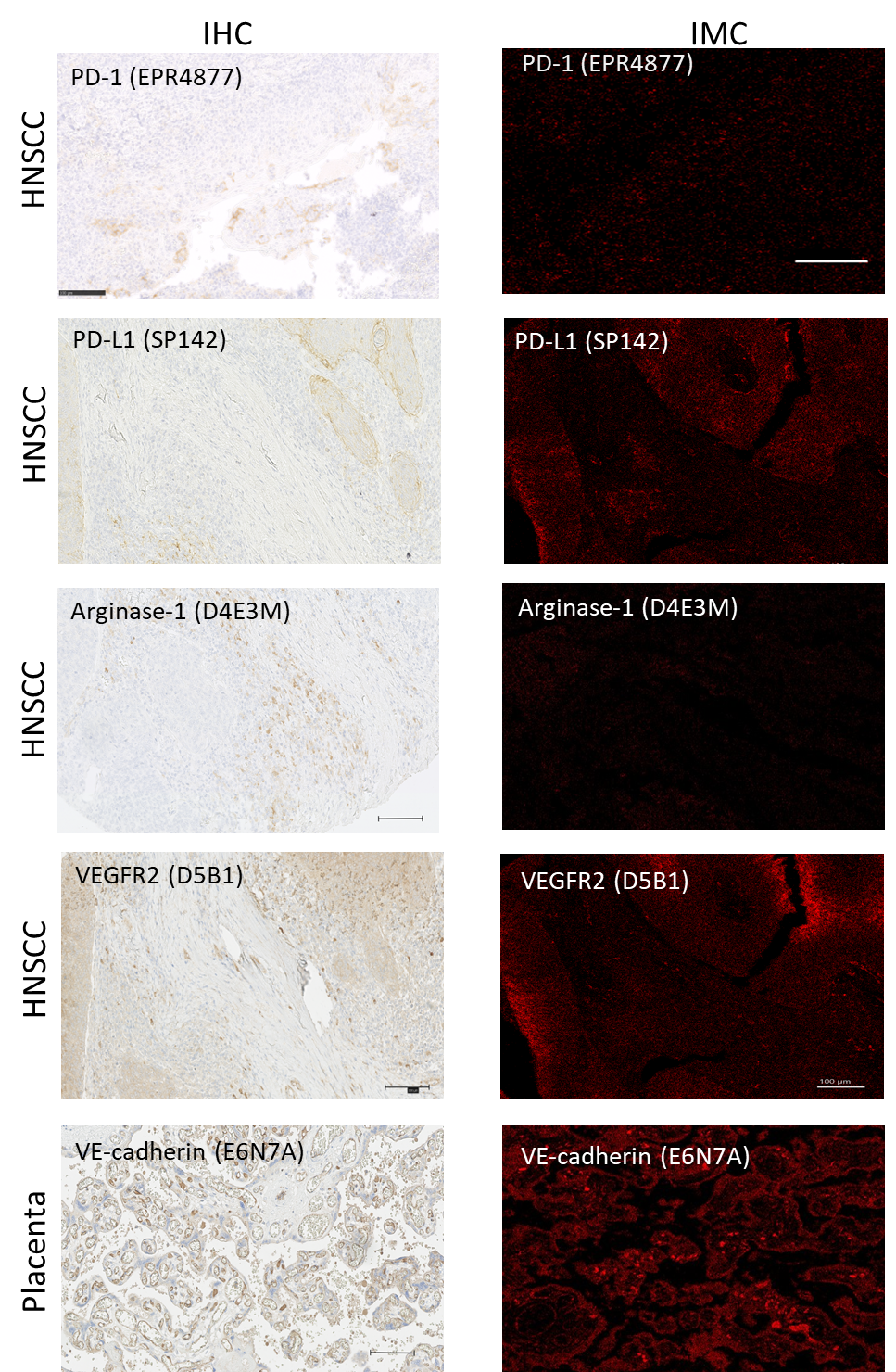


Supplementary figure 2


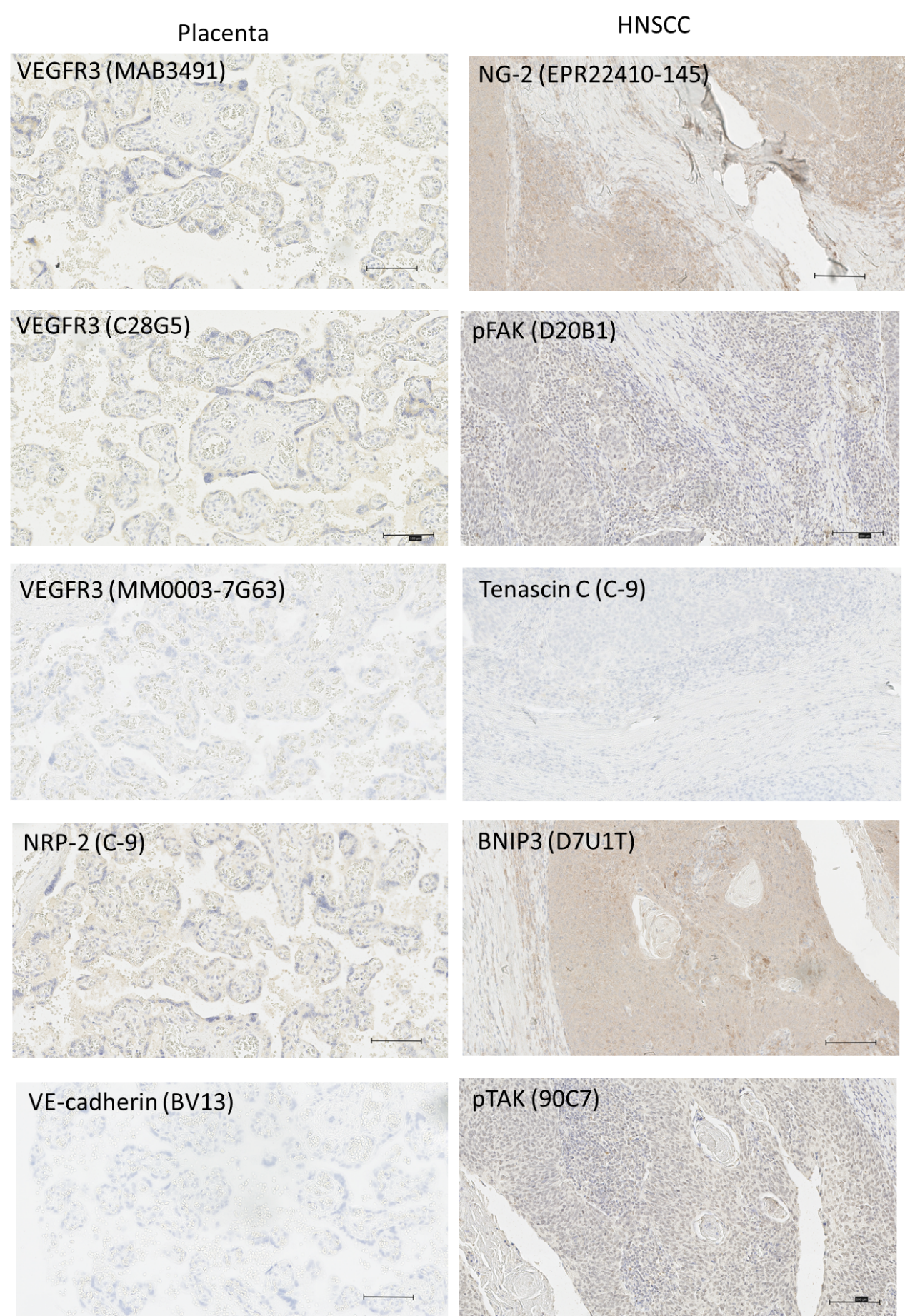


Supplementary figure 3


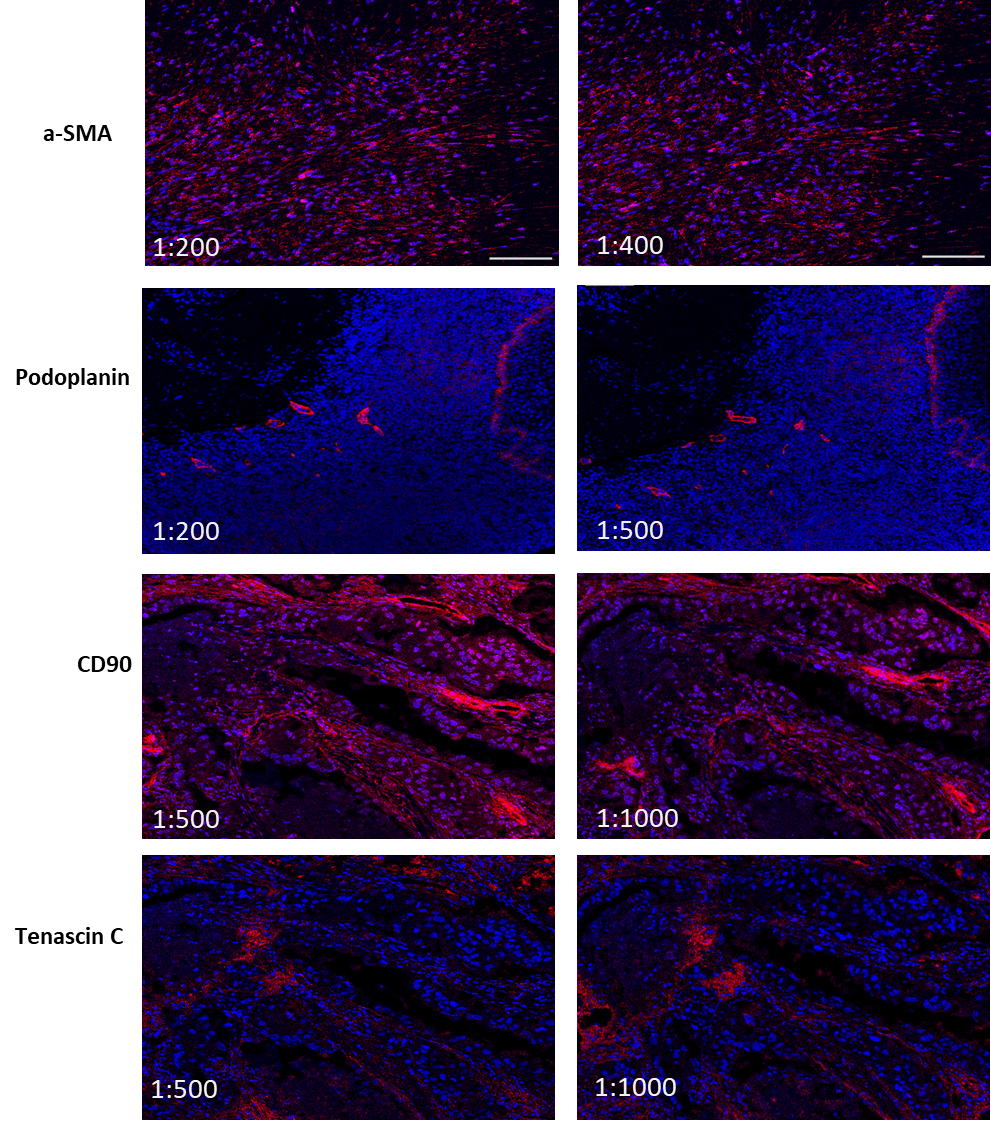


Supplementary figure 4


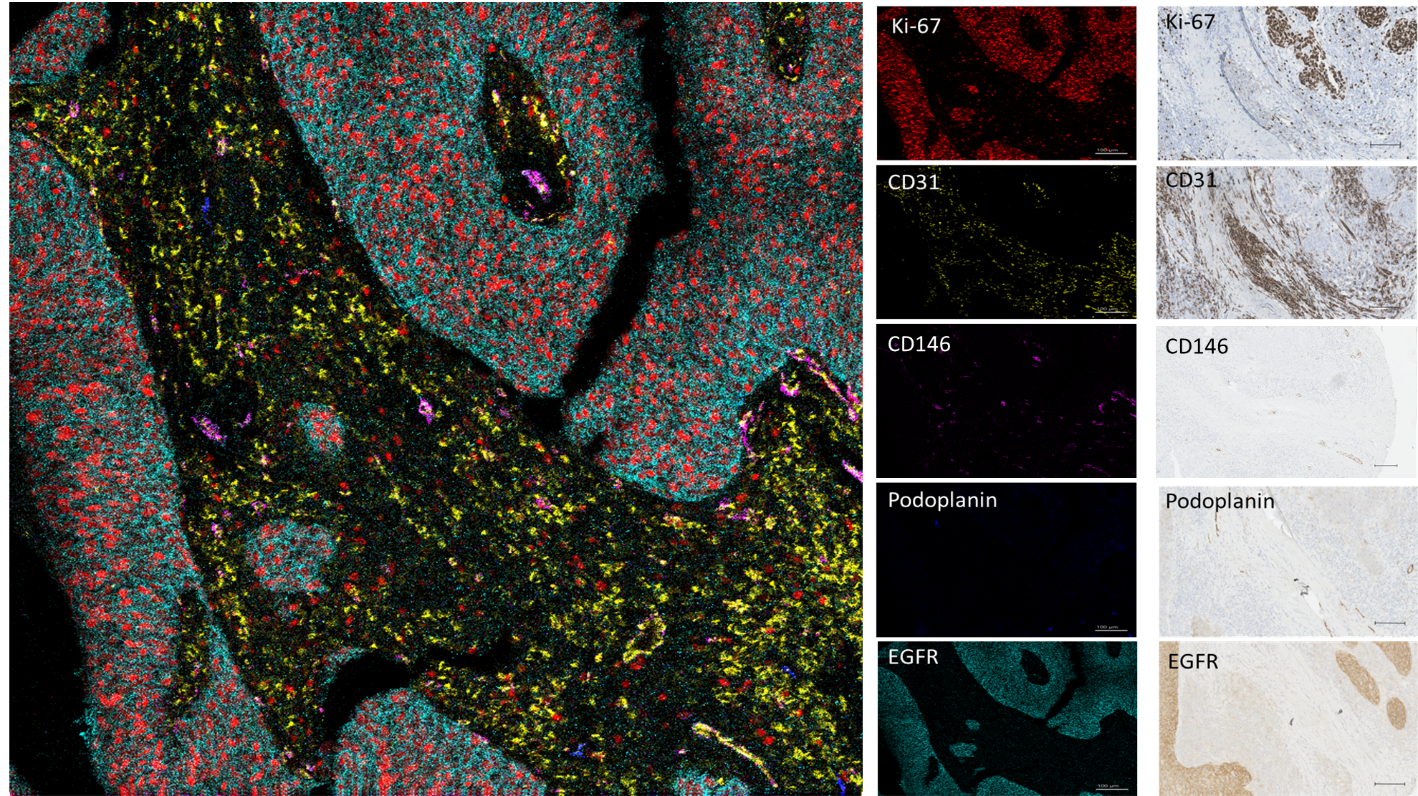


Supplementary figure 5


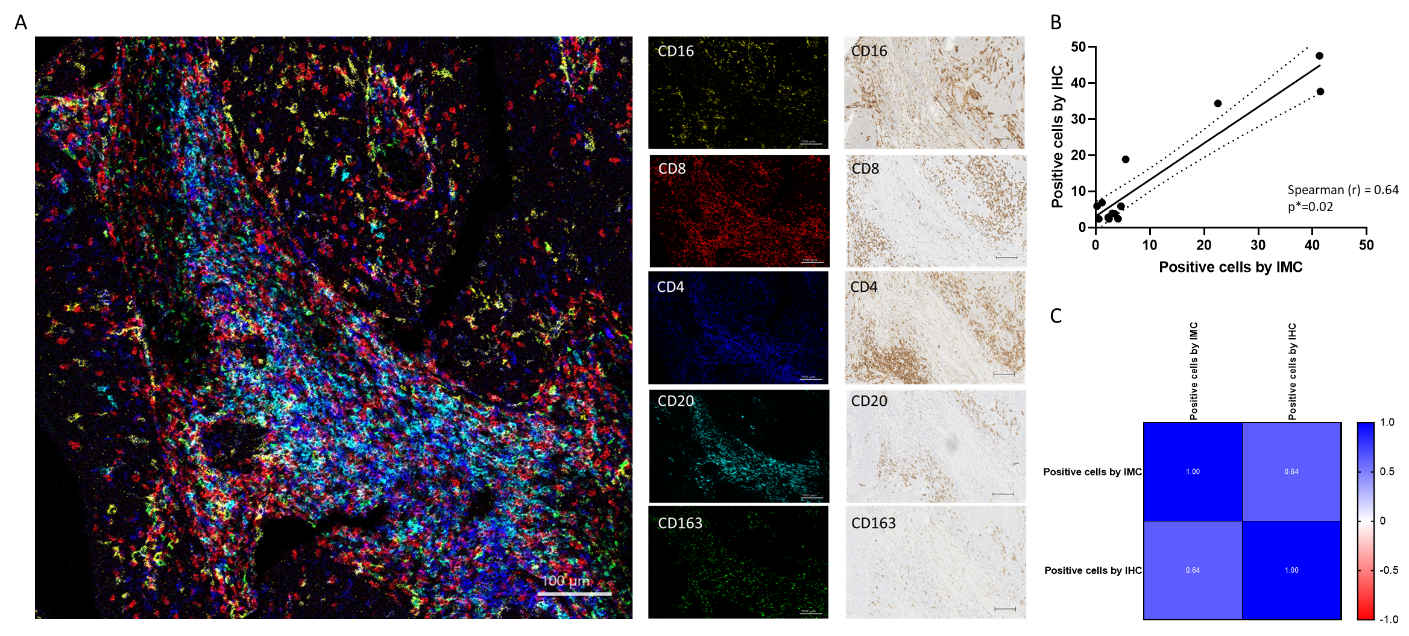


Supplementary figure 6


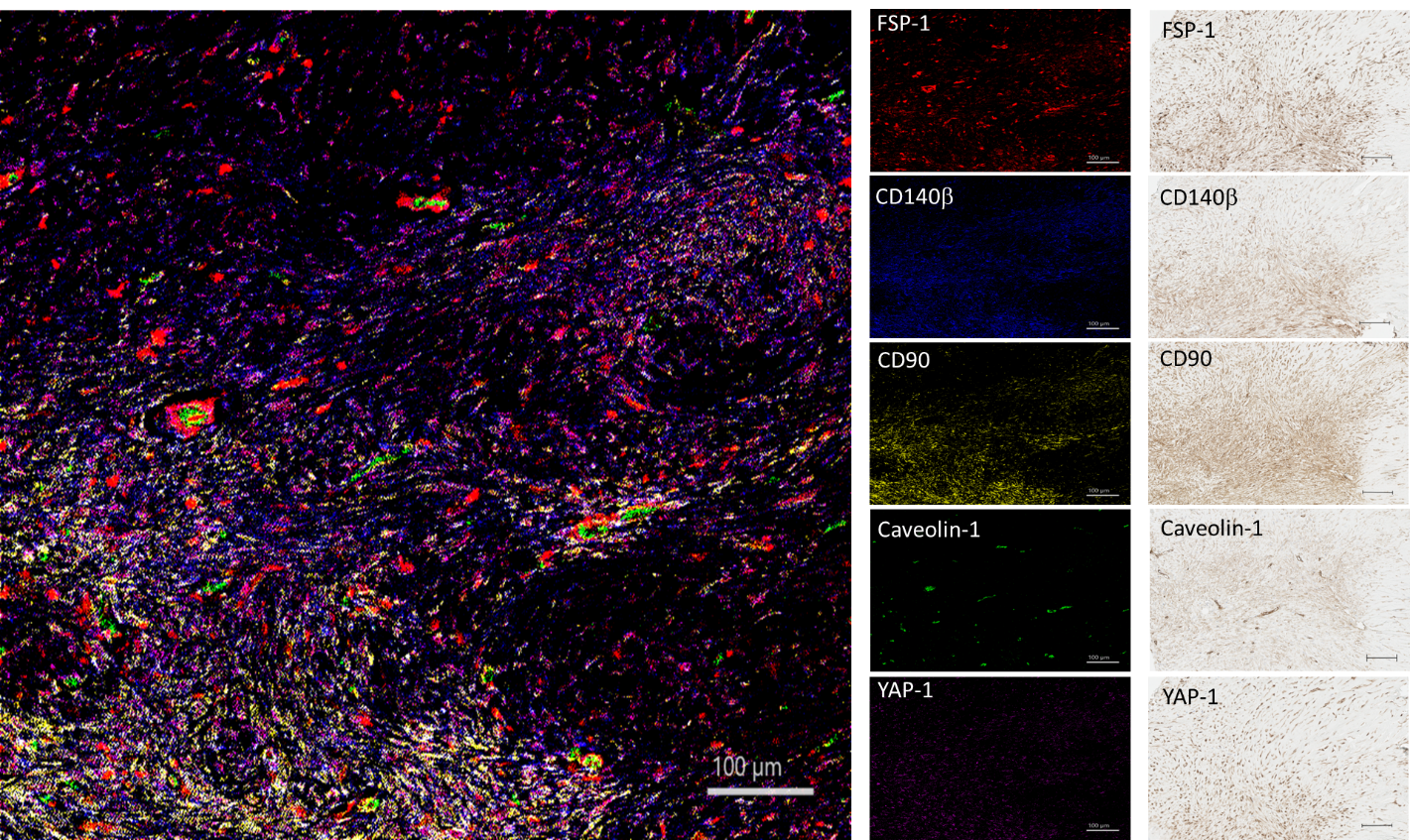


Supplementary figure 7


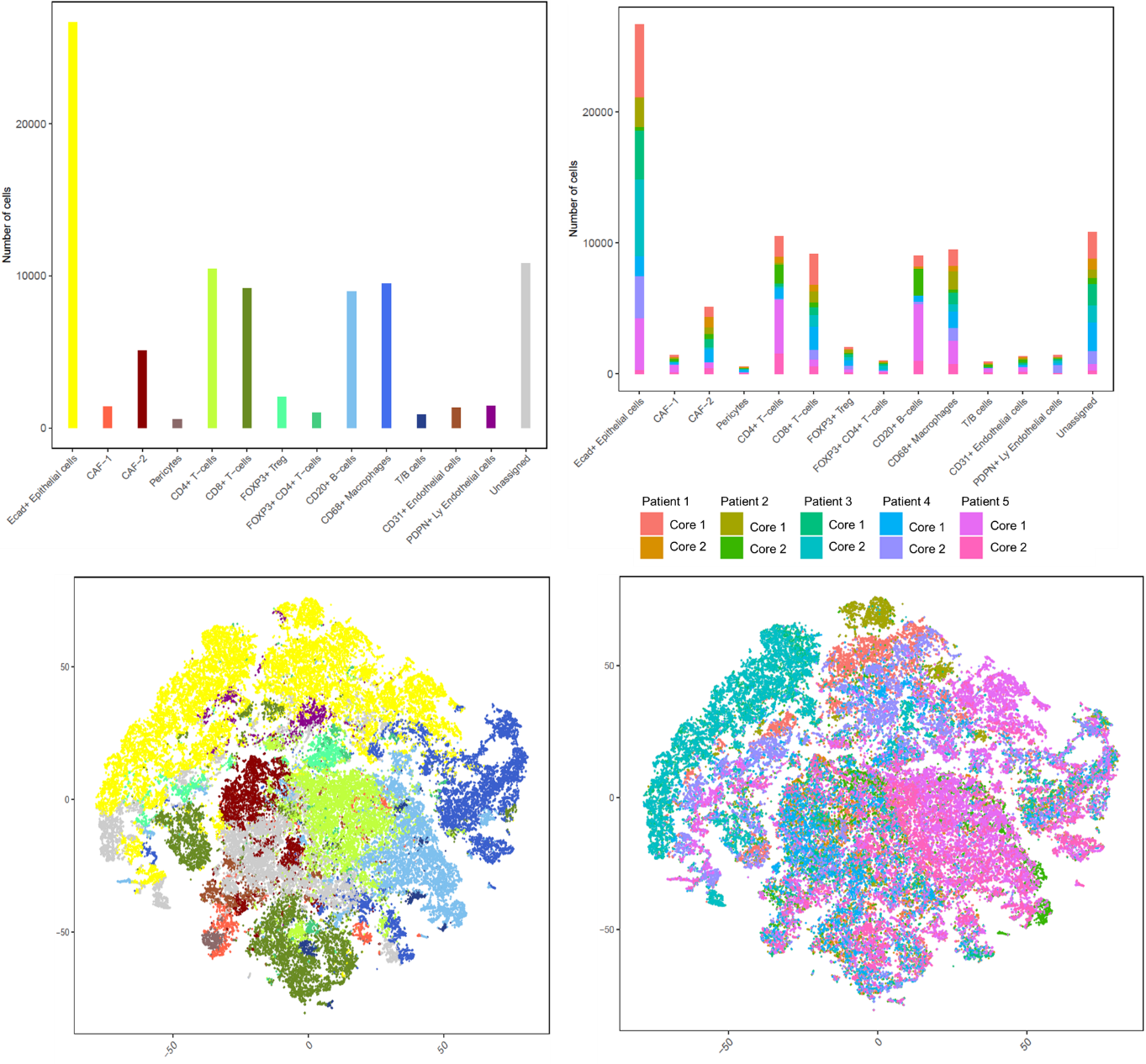

Supplement: Multimedia component 1 [file mmc1.docx]
